# Correction: Poly(adenine)-mediated DNA-functionalized gold nanoparticles for sensitive detection of mercury ions in aqueous media

**DOI:** 10.1039/d1ra90129j

**Published:** 2021-06-28

**Authors:** Jinjin Yin, Jiuchao Wang, Xiyue Yang, Tao Wu, Huashan Wang, Xiaoming Zhou

**Affiliations:** College of Chemical Engineering and Materials Science, State Key Laboratory of Food Nutrition and Safety, Key Laboratory of Food Nutrition and Safety, Tianjin University of Science and Technology Tianjin 300457 China wutaoxx@gmail.com whs@tust.edu.cn

## Abstract

Correction for ‘Poly(adenine)-mediated DNA-functionalized gold nanoparticles for sensitive detection of mercury ions in aqueous media’ by Jinjin Yin *et al.*, *RSC Adv.*, 2019, **9**, 18728–18733, DOI: 10.1039/C9RA03041G.

The authors regret that an incorrect grant number was shown in the Acknowledgements section of the published article. The corrected section should read:

This work is supported by the Natural Science Foundation of Tianjin [No. 18JCQNJC06000] and the Youth Innovation Foundation of Tianjin University of Science and Technology [No. 2016LG16].

The Royal Society of Chemistry apologises for these errors and any consequent inconvenience to authors and readers.

## Supplementary Material

